# Evaluation of Effectiveness of Self-Supervised Learning in Chest X-Ray Imaging to Reduce Annotated Images

**DOI:** 10.1007/s10278-024-00975-5

**Published:** 2024-03-08

**Authors:** Kuniki Imagawa, Kohei Shiomoto

**Affiliations:** Faculty of Information Technology, Tokyo City University, 1-28-1 Tamazutsumi, Setagaya-ku, Tokyo, 158-8557 Japan

**Keywords:** Self-supervised learning, Reduction labeled data, Chest X-ray, COVID-19

## Abstract

A significant challenge in machine learning-based medical image analysis is the scarcity of medical images. Obtaining a large number of labeled medical images is difficult because annotating medical images is a time-consuming process that requires specialized knowledge. In addition, inappropriate annotation processes can increase model bias. Self-supervised learning (SSL) is a type of unsupervised learning method that extracts image representations. Thus, SSL can be an effective method to reduce the number of labeled images. In this study, we investigated the feasibility of reducing the number of labeled images in a limited set of unlabeled medical images. The unlabeled chest X-ray (CXR) images were pretrained using the SimCLR framework, and then the representations were fine-tuned as supervised learning for the target task. A total of 2000 task-specific CXR images were used to perform binary classification of coronavirus disease 2019 (COVID-19) and normal cases. The results demonstrate that the performance of pretraining on task-specific unlabeled CXR images can be maintained when the number of labeled CXR images is reduced by approximately 40%. In addition, the performance was significantly better than that obtained without pretraining. In contrast, a large number of pretrained unlabeled images are required to maintain performance regardless of task specificity among a small number of labeled CXR images. In summary, to reduce the number of labeled images using SimCLR, we must consider both the number of images and the task-specific characteristics of the target images.

## Introduction

Machine learning-based medical image analysis has been researched actively and introduced into the medical environment. This use of such technologies is expected to increase in the future due to the success of deep learning, which is a subset of machine learning [1, 2]. However, a common challenge is the scarcity of medical images due to patient privacy concerns. In addition, the scarcity of labeled medical images is also a serious problem because annotating medical images requires specialized knowledge, and an inappropriate annotation process can introduce annotation bias [3, 4]. To overcome the lack of medical images, the most common approach for supervised learning is to pretrain a convolutional neural network (CNN) on a large number of natural images, e.g., ImageNet [5], and then fine-tune the network using labeled medical images for the target medical task. This approach has proven effective in terms of improving performance in some categories [6]. For example, since the outbreak of coronavirus disease 2019 (COVID-19), several X-ray and CT image datasets have been made available to the public, and many COVID-19 classification studies have demonstrated the effectiveness of this approach, especially for limited images. However, there are significant differences in the pretrained and fine-tuned parameters because ImageNet contains approximately 1.4 million natural color images with 22,000 categories and 1000 labels. Medical applications that utilize CNN models pretrained on ImageNet remain ambiguous and can suffer from model overfitting. In addition, it has been reported that CNN models pretrained on ImageNet can perform worse than models without pretraining, depending on the characteristics of the data [7–9].

Self-supervised learning (SSL) has been proposed recently as an effective approach to the labeled image scarcity problem. The SSL training method produces representations using unlabeled images, and it is a type of unsupervised learning. Generally, the pretrained representation is fine-tuned for a downstream task on a few labeled images. This semi-supervised strategy achieves good performance compared to supervised learning. SimCLR [10] is a simple framework for contrastive learning of visual representations, and its success has led to extensive research on contrastive methods. However, SimCLR requires a large batch size to achieve sufficient performance; thus, many revised contrastive learning frameworks, e.g., Momentum Contrast (Moco) [11] and Bootstrap Your Own Latent (BYOL) [12], have emerged to reduce the batch size. In the medical fields, there has been an increase in the number of studies demonstrating the effectiveness of SSL methods [13]. For example, Shih-Cheng et al. [14] reviewed literature published after 2012 for SSL on medical image classification. They demonstrated the potential of SSL to reduce the amount of labeled data and improve performance and transferability. However, these previous studies demonstrated the effectiveness of SSL by pretraining on a large number of unlabeled images.

In a related study, Shekoofeh et al. [15] demonstrated that pretraining on unlabeled ImageNet and chest X-ray (CXR) images with SimCLR outperformed a supervised method pretrained on ImageNet and improved transferability to dermatology images. In addition, Hari et al. [16] demonstrated that pretraining on CXR images with Moco can reduce the number of labeled images and outperform CXR images without a pretraining process. Guang et al. [17] performed pretraining on labeled ImageNet and then performed pretraining on unlabeled CXR images six SSL methods (Cross, BYOL, SimSiam, SimCLR, PIRL-jigsaw, and PIRL-rotation), which was followed by transfer learning to the target task. They demonstrated the improvement in COVID-19 detection compared to supervised learning and pretraining with SSL methods. These studies also performed pretraining on a large number (in excess of several hundred thousand) of unlabeled CXR images or natural images using SSL.

In real world applications, it would be difficult to prepare such a large number of medical images, even unlabeled images, because utilization of medical data must comply with the laws and regulations of each country. Thus, this study was conducted to confirm the effectiveness of SSL for pretraining on a limited number of unlabeled medical images to reduce the amount of labeled data and improve performance. The methodology employed in this study is similar to that used in various previous studies, where the pretraining representation is fine-tuned for a binary classification. Specifically, we evaluate the number of CXR labeled images with the fine-tuning method (i.e., label fraction) for task-specific and non-task-specific images using supervised learning without pretraining, unsupervised pretraining, and supervised pretraining. Here, a total of 2000 task-specific images and 90,000 non-task-specific images were used for classification of COVID-19. In this paper, the “task-specific” pretraining dataset means that pretraining dataset matches the test dataset primarily in terms of image type, e.g., medical images and natural images, medical images acquired using medical devices, and the target diseases. The results demonstrate that the performance obtained by pretraining on task-specific unlabeled CXR images can be maintained when the number of labeled CXR images is reduced by approximately 40%. In addition, the performance was significantly better than that obtained without pretraining. In contrast, a large number of pretrained unlabeled images are required to maintain performance regardless of task specificity among a small number of labeled CXR images. In summary, to reduce the number of labeled images and improve performance with SSL, we must consider both the number of images and also the task-specific characteristics of the images.

## Material and Methods

### Datasets

In this study, two publicly available CXR datasets were used, i.e., the National Institutes of Health (NIH) dataset [18] and the BrixlA dataset [19]. The NIH dataset contains 112,120 CXR images with 14 diseased and normal images from 30,805 unique patients, and the BrixlA dataset contains 4703 CXR images from COVID-19 patients. Here, a total of 2000 training and validation images for COVID-19 and normal cases were selected randomly at the same ratio because our previous study demonstrated that CNN models, e.g., AlexNet and ResNet, achieve consistent performance when supervised learning is performed on 2000 training images.

The training and validation datasets were divided into labeled images (*N*=100, 250, 500, 1000, 1500, and 2000) and unlabeled images (*N*=1900, 1750, 1500, 1000, 500, and 0) from the NIH and BrixlA datasets. Note that there was no duplication between labeled and unlabeled images. In addition, the 90,000 unlabeled CXR images in the NIH dataset and the 90,000 unlabeled natural images in the ImageNet dataset were selected randomly for training and validation. A test dataset of 1000 CXR images, independent of the training datasets, was used for binary classification of COVID-19. Here, all CXR images were resized to 256 × 256 pixels and cropped around the center to 224 × 224 pixels. The grayscale CXR images were converted from 16-bit to 8-bit and converted to three color channels (red, green, and blue). The pixel values of the input images were normalized between ranges 0 and 1.

### Methodology

**Fig. 1:**
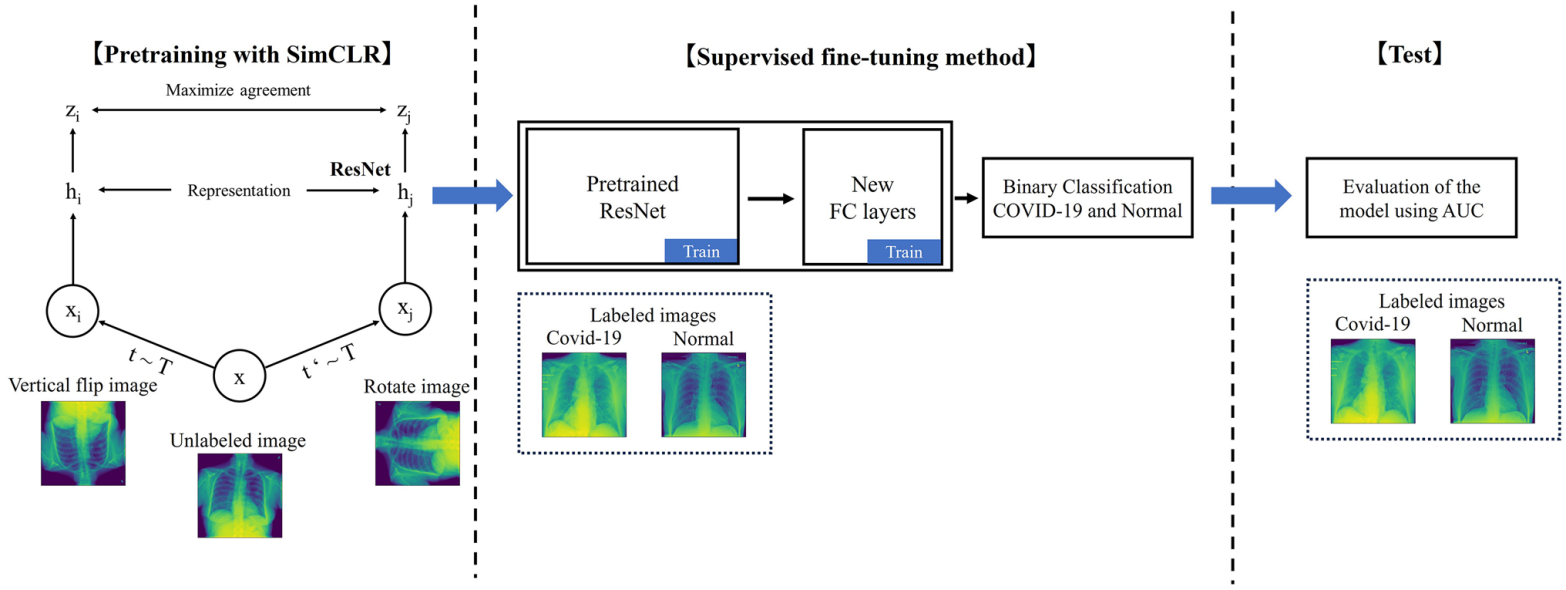
Proposed pretraining process with SimCLR and fine-tuning method for binary classification of COVID-19

In our experiments, we employed SimCLR [10] as the SSL method to learn the visual representations as a pretraining process. Figure 1 shows the method used in our experiments. SimCLR is a simple framework that does not require a special architecture or memory banks. Here, image *x* is differentially augmented $\tilde{\varvec{x}}_i$ and $\tilde{\varvec{x}}_j$ as positive pairs. In addition, f($\cdot$) is an encoder network to generate representations, where $h_i = f(\tilde{\varvec{x}}_i) = ResNet(\tilde{\varvec{x}}_i)$, and g($\cdot$) is a neural network projection head with one hidden layer used for contrastive loss, where $z_i = g(h_i) = W^{(2)}\sigma (W^{(1)}\varvec{h}_i)$. *N* is an arbitrary number of batches with 2*N* positive pairs in each batch and 2$(N-1)$ negative pairs. SimCLR maximizes the agreement of the positive pairs and minimizes the negative pairs using the contrasting loss as follows:

1 $$\begin{aligned} \ell _{i,j} = -\log \frac{\textrm{exp}(\textrm{sim}(\varvec{z_i}, \varvec{z_j})/\tau )}{\sum _{k=1}^{2N} \parallel _{[k \ne i]}\exp (\textrm{sim}(\varvec{z_i}, \varvec{z_k})/\tau )} \end{aligned}$$

where $\tau$ is a temperature parameter, and $\parallel$ means if $k= i$ then 0 and $k \ne i$ then 1. In addition, sim() denotes the cosine similarity, and *N* is the batch size.

We performed some experiments to select the type of data augmentation, the backbone network, and the hyperparameters such as the number of batches and the number of training epochs, because the original paper [10] reported that these have a significant impact on performance. For data augmentation technique, two types of transformations were used in this evaluation, i.e., spatial transformations, e.g., cropping, rotation, vertical flipping, and horizontal flipping, and appearance transformations, e.g., Gaussian blur and grayscale. In addition, different numbers of layers (*N* = 18, 34, 50, and 101) in the ResNet backbone were also evaluated, and we investigated different numbers of batches (*N* = 32, 64, 128, and 256) and training epochs (*N* = 100, 300, and 500). Note that other SimCLR hyperparameters were unchanged, and the hyperparameters for supervised learning were based on our previous results [20]. The area under the curve (AUC) was used for evaluation. The sensitivity and specificity depend on the classification threshold, and accuracy is dependent on the proportion of the test dataset. The 95% confidence interval was constructed using the method proposed by Hanley and McNeil method [21]. To evaluate the effectiveness of the SSL, the AUC on the common test dataset was determined as a function of pretraining on unlabeled images and the number of labeled CXR images for the subsequent supervised learning process. The types and number of images used for each training and test are shown in Table 1.

**Table 1:** Types and number of images used for training and test

Number of labeled CXR images with supervised fine-tuning method			100	250	500	1000	1500	2000
(NIH and BrixlA dataset)								
Method	Image type	Dataset	Number of pretraining images					
No pretraining	-	-	0	0	0	0	0	0
Unsupervised pretraining (SimCLR)	CXR images^a^	NIH and BrixlA	1900	1750	1500	1000	500	0
	CXR images^b^	NIH	90000	90000	90000	90000	90000	0
	Natural images	ImageNet	90000	90000	90000	90000	90000	0
Supervised pretraining (ResNet34)	Natural images	ImageNet	1400000	1400000	1400000	1400000	1400000	0
Text	CXR images	NIH and BrixlA	1000	1000	1000	1000	1000	1000

^a^The images are identical to the test images in terms of the dataset and the target disease

^b^The images are not included target disease

## Results

**Fig. 2:**
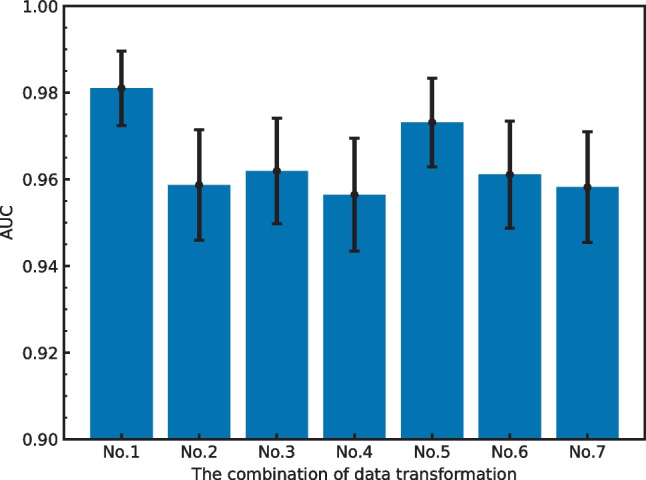
AUC versus the combination of transformations. No. 1: rotate and vertical; No. 2: rotate and horizontal flip; No. 3: rotate and crop; No. 4: rotate and Gaussian blur; No. 5: rotate and grayscale; No. 6: crop and vertical flip; No. 7: Gaussian blur and grayscale. SimCLR (500 images) and ResNet34 (1500 images) with batch sizes of 128 and 300 epochs were used in this evaluation

**Fig. 3:**
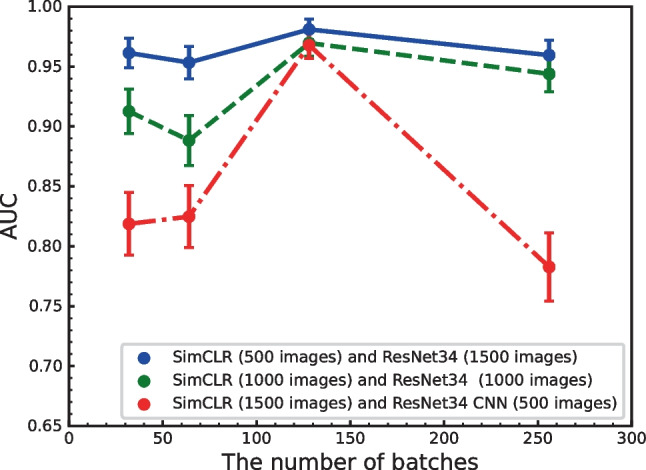
AUC versus the number of batches (*N*= 32, 64, 128, and 256). Here, a combination of rotation and vertical flip was used over 300 epochs

**Fig. 4:**
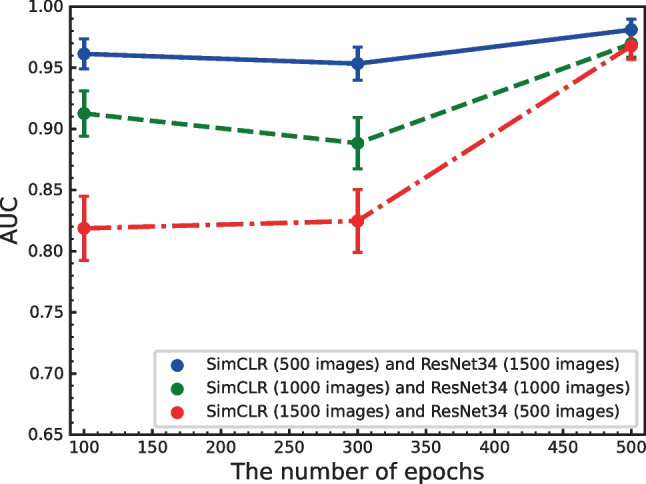
AUC versus the number of epochs (*N*= 100, 300, and 500). A combination of rotation and vertical flip was used with a batch size of 128

**Fig. 5:**
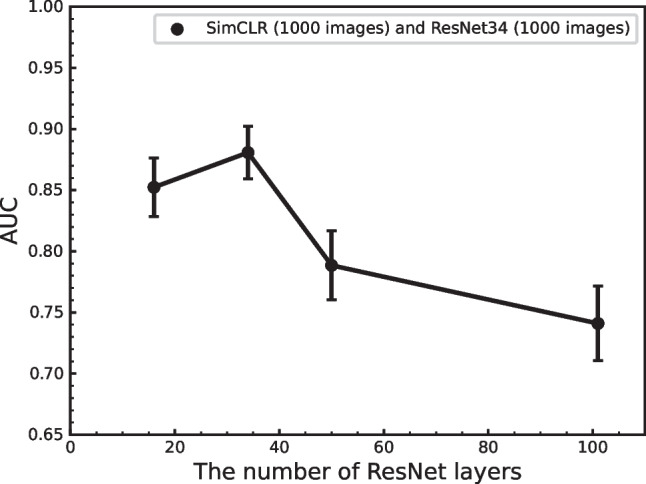
AUC versus the number of ResNet layers (*N*= 18, 34, 50, and 101). A combination of rotation and vertical flip was used over 300 epochs with a batch size of 128

Figure 2 shows the performance obtained using these enhancements when applied in various combinations. We found that the combination of rotation and vertical flipping obtained the highest performance. Figures 3 and  4 show the effect of batch size and the number of training epochs on performance. As can be seen, the best performance was obtained with a batch size of 128. In addition, higher numbers of training epochs tended to improve performance; however, but 300 epochs were used due to computational resources. We also evaluated the effect of different numbers of ResNet layers. ResNet34 was the most effective deep layer compared to other layers in Fig. 5. Note that the fundamental model and hyperparameter configurations were the same for all experiments (see “Methodology”), and the detailed experimental conditions are shown in Figs. 2,  3,  4 and  5.

**Fig. 6:**
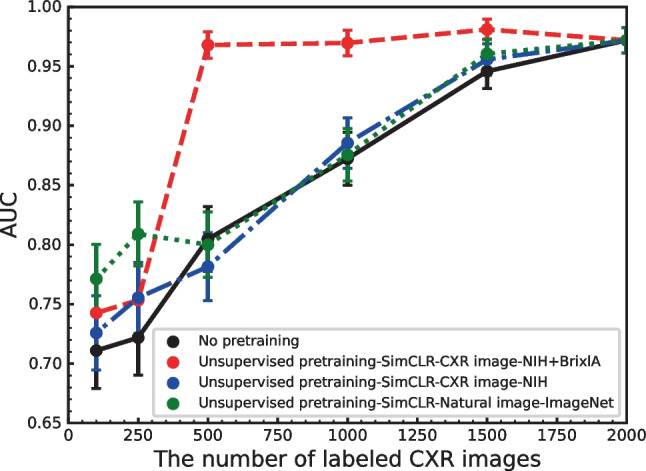
AUC versus the number of labeled CXR images with the supervised fine-tuning method used by supervised learning without pretraining and unsupervised pretraining process. The numbers of images for each training and test are shown in Table 1

The pretrained representations with different types and numbers of images were subsequently fine-tuned on different numbers of labeled CXR images. Figure 6 shows AUC versus the number of CXR labeled images with the fine-tuning method for each unsupervised method pretrained on unlabeled images. Note that Fig. 6 includes several task-specific CXR images extracted from the NIH and BrixlA datasets (red), 90,000 CXR images extracted from only the NIH dataset (blue) and 90,000 natural images extracted from ImageNet (green). The supervised learning without pretraining was also included as a baseline method (black). We found that the AUCs for pretraining on task-specific CXR images can be maintained when the number of labeled CXR images is reduced from 2000 to 500 (i.e., a reduction of approximately 40%). In addition, performance was significantly better than the supervised baseline in this area. In contrast, the effect of SimCLR was reduced drastically when the number of labeled images was reduced to less than 500. When pretraining was performed on 90,000 non-task-specific CXR images and 90,000 natural images, we observed slightly better performance compared to the supervised baseline. In contrast, the improvement in terms of the effectiveness of SimCLR can be observed in the small labeled image region.

## Discussion

The implementation of SimCLR on ImageNet requires large batch sizes; thus, this approach consumes significant computational resources. However, our results demonstrate that SimCLR specific to the CXR images did not require a large batch size. We assume that the CXR images have common anatomical structures across the images; thus, so there is no need to create many negative pairs in each batch to reduce the loss function. The results of data augmentation and the backbone network are also specific to the CXR images. As a backbone, we found that ResNet does not require deeper layers, and this trend is similar to the supervised learning results presented by D’souza et al. [22]. They suggested that “deeper is better” is not always true, especially for small amounts of data, and the optimal CNN network depends on the nature of the training data. This suggests that feature extraction with SimCLR does not necessarily require a deep model because CXR images are simpler than ImageNet and the number of images is very small. In addition, from a computational resource perspective, our results also demonstrate that it is important to use SimCLR with task-specific images rather than ImageNet.

In this study, SimCLR was used specifically for CXR images, and we found that the AUCs of the pretrained task-specific CXR images can be maintained when the number of labeled CXR images is reduced by approximately 40%. Kyungjin et al. [23] also investigated the effectiveness of an SSL method and demonstrated the performance for some CXR datasets, e.g., the CheXpert datasets [24] with fine-tuning method for multiclassification used by pretraining on non-task-specific 4.8 million unlabeled CXR images with Moco. Table 2 compares these results in terms of the reduction of fine-tuned labeled images. Although performing direct quantitative comparisons is difficult due to the differences in the type of SSL method, the number of classifications, and the number of labeled images, the results demonstrate a similar trend. In other words, AUCs can be maintained when the number of labeled CXR images is reduced by approximately half. Considering that the number of unlabeled pretraining images used in the current study was extremely small (ranging from hundreds to thousands), the acquisition of task-specific images is an important factor in terms of data efficiency.

**Fig. 7:**
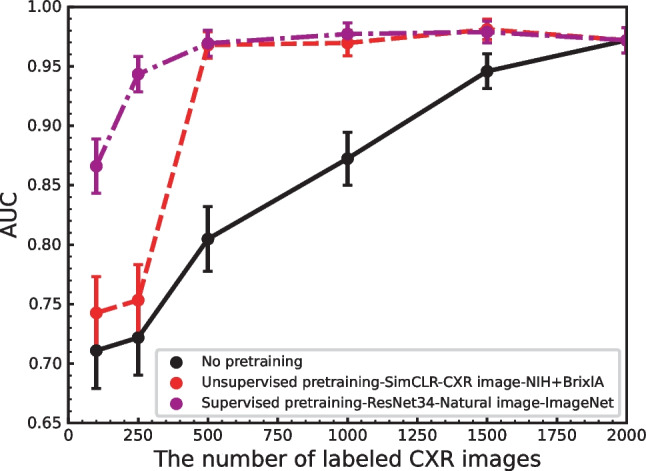
AUC versus the number of labeled CXR images with the supervised fine-tuning method used by supervised learning without pretraining, unsupervised pretraining and supervised pretraining. The numbers of images used for each training and test are shown in Table 1

In contrast, the AUC performance obtained when pretraining with task-specific images demonstrated a significant reduction compared to the performance obtained with non-task-specific images among a small number of labeled CXR images. Figure 7 shows the number of CXR labeled images with the fine-tuning method for both unsupervised and supervised methods pretrained on unlabeled and labeled images. This figure includes task-specific unlabeled CXR images extracted from the NIH and BrixlA datasets (red) and the labeled natural images extracted from ImageNet (purple) as pretraining. Note that the supervised learning without pretraining is also included as a baseline method (black). As shown, pretraining on 1.4 million labeled ImageNet clearly outperforms in small labeled image regions (less than 500 images). A related study by Sellergren et al. [25] achieved an AUC comparable to state-of-the-art deep supervised learning models on tens to hundreds of labeled images by performing pretraining on 821,544 unlabeled CXR images using the SSL method. This suggests that a large number of images is required to maintain sufficient performance regardless of the pretraining method and task specificity in small labeled image regions.

In this study, we found that the performance obtained by pretraining on the task-specific unlabeled images with SimCLR can reduce the number of labeled images and outperform the process that does not employ pretraining. However, this result is limited in that does not apply to a small number of labeled images. Recently, many studies have reported the effectiveness of SSL; however, to the best of our knowledge, few studies have investigated that use of a small number of labeled and unlabeled images that reflect the real world. Thus, the results of the current study provide fundamental insight into the effectiveness of SSL in the medical field. Note that our study is limited in terms of the generalizability of our findings. First, the experiment conducted in this study only investigated SimCLR; thus, other improved and refined SSL frameworks should be considered in the future. In addition, more detailed hyperparameters should be considered. Second, a wider variety of diseases should be considered to the reflect actual clinical practices. For COVID-19 detection, similar diseases, e.g., viral and bacterial pneumonia, should be investigated. In addition, other medical images should also be considered.

**Table 2:** Fraction of labeled images

Label Fraction (%)	1	5	10	12.5	50	100
Ours	N/A	0.742	N/A	0.753	0.970	0.972
Kyungjin et al. [23]	0.638	N/A	0.746	N/A	0.790	0.807

## Conclusion

To the best of our knowledge, this paper represents the first report demonstrating the effectiveness of the SSL method with pretraining on a small number of unlabeled images. We hope that the results of this study will contribute to the ongoing development of machine learning-based medical image analysis.

## Data Availability

The NIH dataset and the BrixlA dataset that support the findings of this study are openly available at https://nihcc.app.box.com/v/ChestXray-NIHCC and https://brixia.github.io/.
